# Extended-Spectrum β-Lactamase-Producing *Klebsiella pneumoniae* in Dogs from Cape Verde and São Tomé and Príncipe: Implications for Public Health

**DOI:** 10.3390/antibiotics14040408

**Published:** 2025-04-16

**Authors:** Raquel Abreu, Alice Matos, Luís Capela, Rita Jorge, Joana F. Guerreiro, Gonçalo Pereira, Eva Cunha, Lélia Chambel, Luis Tavares, Filip Boyen, Manuela Oliveira

**Affiliations:** 1 CIISA—Centre for Interdisciplinary Research in Animal Health, Faculty of Veterinary Medicine, University of Lisbon, 1300-477 Lisbon, Portugaljguerreiro@fmv.ulisboa.pt (J.F.G.); goncalopereira@fmv.ulisboa.pt (G.P.); evacunha@fmv.ulisboa.pt (E.C.); ltavares@fmv.ulisboa.pt (L.T.); moliveira@fmv.ulisboa.pt (M.O.); 2 AL4AnimalS—Associate Laboratory for Animal and Veterinary Sciences, 1300-477 Lisbon, Portugal; 3 Associação Veterinários Sem Fronteiras, 1300-477 Lisboa, Portugal; malice.omatos@gmail.com (A.M.);; 4 BioISI—BioSystems & Integrative Sciences Institute, Faculty of Sciences, University of Lisbon, 1749-016 Lisbon, Portugal; lmchambel@ciencias.ulisboa.pt; 5 Faculty of Veterinary Medicine, Department of Pathobiology, Pharmacology and Zoological Medicine, Ghent University, 9000 Merelbeke, Belgium; filip.boyen@ugent.be; 6 cE3c—Centre for Ecology, Evolution and Environmental Changes & CHANGE—Global Change and Sustainability Institute, Faculty of Sciences, University of Lisbon, 1749-016 Lisbon, Portugal

**Keywords:** dog, *Klebsiella pneumoniae*, antimicrobial resistance, ESBL, multidrug resistance, Cape Verde, São Tomé and Príncipe

## Abstract

Antimicrobial resistance is a growing global threat, with surveillance providing essential information to control its spread and support rational treatment strategies. *Klebsiella pneumoniae*, a member of the Gram-negative Enterobacteriaceae family, frequently develops resistance mechanisms. This study analyzed 195 rectal swabs from companion and stray dogs in Santiago and São Nicolau (Cape Verde) and São Tomé and Príncipe, sampled during a neutering and deworming campaign conducted by Veterinary Without Borders Portugal, to detect extended-spectrum β-lactamase (ESBL)-producing bacteria. Samples were enriched and then cultured on ChromID^®^ ESBL agar, and resulting isolates were identified via MALDI-TOF MS. A total of 35 *K. pneumoniae* isolates were identified, of which 32 were confirmed as ESBL producers. Antimicrobial susceptibility testing showed 100% resistance to aztreonam, cefotaxime, cefpodoxime, and ceftaroline, and high resistance to cefepime (93.8%), ciprofloxacin (93.8%), and trimethoprim/sulfamethoxazole (90.6%). All isolates were considered multidrug-resistant but remained susceptible to cefoxitin, imipenem, and meropenem. The genes *bla*_CTX-M_, *bla*_SHV_, and *bla*_TEM_ were present in 96.9%, 65.6%, and 56.3% of the isolates, respectively. DNA fingerprinting revealed seven clusters, suggesting genetic diversity and strain dissemination across locations. These findings highlight the role of dogs as vectors for antimicrobial resistance dissemination, underscoring the need for continuous surveillance in both veterinary and human medicine.

## 1. Introduction

Antimicrobial resistance (AMR) is a growing public health concern involving the human, animal, and environmental sectors, associated with higher morbidity and mortality rates and a high economic impact [1]. Among the various emerging resistance mechanisms, the production of β-lactamases by several bacterial species is one of the most significant. Members of the Enterobacteriaceae family, such as *Klebsiella pneumoniae* and *Escherichia coli*, are the primary bacteria associated with β-lactamase production, posing a serious healthcare challenge worldwide [2]. The most epidemiologically relevant group of β-lactamases is the extended-spectrum β-lactamase (ESBL) group, which has become endemic worldwide [3].

The classification of β-lactamase enzymes is based on either their primary structure, molecular classes A, B, C, and D, or functional characteristics [4]. Class A enzymes are the most predominant and important source of both natural and acquired resistance to β-lactams, particularly in Enterobacteriaceae [5]. Group A β-lactamases include ESBL enzymes, which confer resistance to most β-lactam antibiotics, including oxyimino-cephalosporins and monobactams [3]. Most ESBLs originate from the TEM (Temoniera) and SHV (sulfhydryl variable) β-lactamases; however, CTX-M (cefotaximase) ESBLs have been rapidly disseminating among Enterobacteriaceae [6]. Most CTX-M enzymes can be clustered into five groups based on sequence homologies: CTX-M-1, CTX-M-2, CTX-M-8, CTX-M-9, and CTX-M-25 [3,4]. The rapid and widespread dissemination of CTX-M ESBLs is significantly altering the epidemiology of ESBL-producing bacteria. These enzymes have become dominant and are now the most prevalent ESBLs in Enterobacteriaceae [3], underscoring the urgent need for global surveillance within the One Health approach.

Surveillance programs primarily focus on the food chain, which constitutes an important route for the spread of antimicrobial-resistant bacteria. To address this, the World Health Organization (WHO) developed the Global Tricycle Surveillance protocol, which includes monitoring AMR across human health, food production, and environmental sectors, and could be implemented in low-resource settings [7].

Beyond food-related transmission, companion animals also play a significant role in AMR dissemination [8]. They share both indoor and outdoor environments with humans [9,10], and frequently circulate near areas occupied by humans, particularly in developing countries. This proximity may contribute to the exposure of humans to dogs’ fecal matter, which could serve as a source of antibiotic resistance transmission among animals and people in areas where sanitation measures are not properly addressed [11]. Consequently, this dynamic facilitates the spread of antimicrobial-resistant bacteria across all One Health sectors [9,10]. In addition, stray animals could act as efficient AMR reservoirs for this dissemination, representing an epidemiological link between humans, livestock, wild animals, and natural environments [12]. The frequent detection of multidrug-resistant (MDR) bacteria in these animals worldwide highlights their role in the One Health AMR framework.

Within surveillance protocols, ESBL-producing *E. coli* is frequently selected as the sentinel pathogen due to its ubiquity and role in resistance transmission [13]. However, this approach may not fully capture the broader picture, as *K. pneumoniae* is also globally widespread and one of the most prevalent ESBL-producing bacteria along with *E. coli* [14]. Being a member of the Enterobacteriaceae family, *K. pneumoniae* is commonly found in environmental settings like soil, water, and sewage, as well as in the intestinal tracts of animals and humans [15]. Despite its widespread presence, *K. pneumoniae* is a significant opportunistic pathogen, being frequently responsible for opportunistic and healthcare-associated infections in both humans and animals, including dogs [16,17]. Treating *K. pneumoniae* infections is often challenging due to the emergence of antibiotic-resistant strains, which are associated with high morbidity and mortality rates [18]. This pathogen is part of the ESKAPE group (*Enterococcus faecium*, *Staphylococcus aureus*, *Klebsiella pneumoniae*, *Acinetobacter baumannii*, *Pseudomonas aeruginosa*, and *Enterobacter* species), a group of bacteria that are the leading cause of hospital-acquired infections worldwide [19]. Additionally, ESBL- and carbapenemase-producing *K. pneumoniae* strains are classified in the critical priority group of pathogens on the WHO bacterial priority pathogens list [20], highlighting the need for enhanced surveillance and control measures across the various affected sectors.

However, many low-resource settings, including some African countries, face significant challenges in combating AMR due to inadequate healthcare infrastructures, a shortage of technical expertise, and a lack of essential supplies for proper diagnosis and treatments, which are often based on presumptive clinical diagnosis, with antibiotics prescribed empirically. Additionally, weak regulations lead to a lack of enforcement of the prescription-only dispensing of antibiotics, leading to the widespread availability of over-the-counter drugs, contributing to the uncontrolled availability and use of antibiotics across the African continent [21], consequently contributing to AMR dissemination [22,23]. As a result, infectious diseases and AMR rates continue to rise in low- and middle-income countries (LMICs), including Cape Verde and São Tomé and Príncipe. Also, AMR surveillance in Africa remains inadequate, facing challenges in establishing efficient surveillance systems and collecting comprehensive AMR data [24], with only eight countries having reported AMR data in the last Global Antimicrobial Resistance Surveillance System (GLASS) report on the implementation of national surveillance systems and AMR rates [25]. Despite GLASS, weak policy implementation and poor coordination contribute to significant gaps in worldwide AMR monitoring. Addressing these topics is crucial to mitigate the spread of resistance and improve public health outcomes.

Consequently, AMR remains a critical global health threat, particularly in LMICs [23], and data regarding ESBL-producing Enterobacteriaceae prevalence in the African region are lacking [1]. Most studies on the African continent have focused on human clinical and community settings [26,27], while no data on the molecular epidemiology of ESBL-producing bacteria from animals have been reported in Cape Verde and São Tomé and Príncipe. In this study, fecal samples were used for surveilling MDR ESBL-producing *K. pneumoniae* carriage in dogs. By doing so, this research aims to provide insights into the distribution of resistance genes among host species in a One Health context.

## 2. Results

### 2.1. Bacterial Isolation and Identification

A total of 35 isolates of presumptive ESBL-producing *K. pneumoniae* were collected from 32 fecal samples (16.4%; n = 32/195); 8.6% (n = 3) of the isolates were obtained from samples from São Nicolau, 8.6% (n = 3) from São Tomé, 20.0% (n = 7) from Santiago, and 62.9% (n = 22) from Príncipe islands.

All 35 presumptive isolates were identified as *K. pneumoniae* by MALDI-TOF MS, with scores ranging from 2.09 to 2.57.

Among the 35 isolates obtained, 32 were phenotypically confirmed to be ESBL-producing *K. pneumoniae*. Regarding the specificity of the culture medium used for screening, 91.4% (n = 32) of the isolates obtained were correctly phenotypically identified as ESBL producers.

### 2.2. Antimicrobial Susceptibility Testing

**Figure 1:**
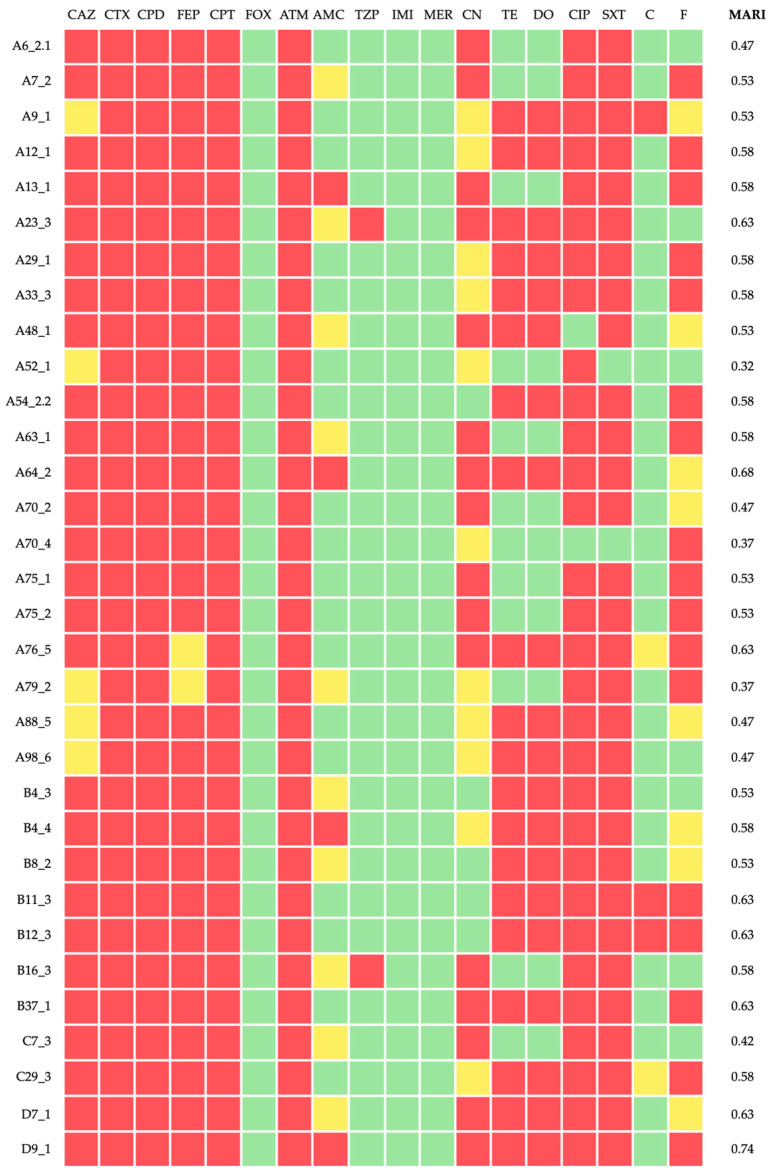
Antimicrobial susceptibility profile and MAR index of each *K. pneumoniae* isolate. The color code indicates resistance (red), intermediate (yellow), and susceptibility (green) against the respective antimicrobial compound. CAZ—ceftazidime; CTX—cefotaxime; CPD—cefpodoxime; FEP—cefepime; CPT—ceftaroline; FOX—cefoxitin; ATM—aztreonam; AMC—amoxicillin/clavulanic acid; TZP—piperacillin/tazobactam; IMI—imipenem; MER—meropenem; CN—gentamicin; TE—tetracycline; DO—doxycycline; CIP—ciprofloxacin; SXT—trimethoprim/sulfamethoxazole; C—chloramphenicol; F—nitrofurantoin; MARI—MAR index.

The resistance rates of the 32 ESBL-producing isolates ranged between 0.0% and 100.0%, as follows: 100.0% (n = 32) were resistant to cefotaxime, cefpodoxime, ceftaroline, and aztreonam; 93.8% (n = 30) to cefepime and ciprofloxacin; 90.6% (n = 29) to trimethoprim/sulfamethoxazole; 84.4% (n = 27) to ceftazidime; 62.5% (n = 20) to tetracycline and doxycycline; 53.1% (n = 17) to nitrofurantoin; 50.0% (n = 16) to gentamicin; 12.5% (n = 4) to amoxicillin/clavulanate; and 9.4% (n = 3) to piperacillin/tazobactam and chloramphenicol. All isolates were susceptible to cefoxitin, imipenem, and meropenem (Table 1, Figure 1). All isolates were considered MDR, according to [28]. MAR indices ranged between 0.42 and 0.74 (Figure 1).

No significant association was found between the MAR index of the isolates and the ownership status of the sampled dogs (*p* = 0.46).

### 2.3. Identification of β-Lactamase Genes by PCR

**Figure 2:**
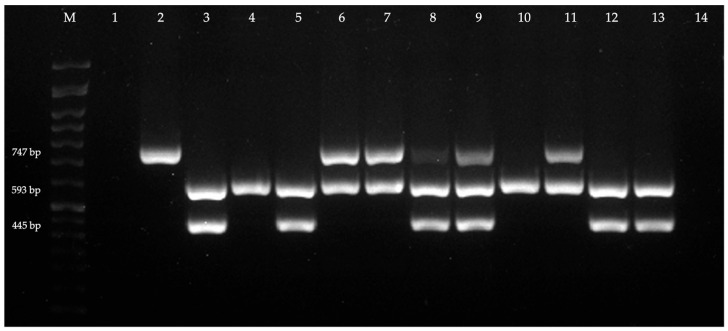
PCR amplification of *bla*_CTX-M_, *bla*_TEM_, and *bla*_SHV_. M—molecular weight marker; 1—*E. coli* ATCC 25922, negative for the presence of the screened genes; 2—*K. pneumoniae* CECT 7787, producing SHV (747 bp); 3—*E. hormaechei* subsp. *hoffmannii* CCUG 58962, producing CTX-M (593 bp) and TEM (445 bp); 4 and 10—isolates producing CTX-M; 5, 12, and 13—isolates producing CTX-M and TEM; 6, 7, and 11—isolates producing CTX-M and SHV; 8 and 9—isolates producing SHV, CTX-M, and TEM; 14—negative control (PCR-grade water).

All 32 *K. pneumoniae* isolates displaying an ESBL phenotype demonstrated the presence of at least one *bla* gene (Figure 2). The presence of the genes *bla*_CTX-M_, *bla*_SHV_, and *bla*_TEM_ was detected in 96.9% (n = 31), 65.6% (n = 21), and 56.3% (n = 18) of the isolates, respectively (Table 2). Globally, 21.9% (n = 7) of the isolates were positive for the presence of one gene, 37.5% (n = 12) for the presence of two genes, and 40.6% (n = 13) for the presence of three genes. It was possible to observe several types of patterns, namely, of individual genes associated with the production of CTX-M (18.8%; n = 6) and SHV (3.1%; n = 1); of the simultaneous presence of two genes encoding for ESBLs, including CTX-M + TEM (15.6%; n = 5) and CTX-M + SHV (21.9%; n = 7); and of the presence of three genes encoding for ESBLs, namely, CTX-M + TEM + SHV (40.6%; n = 13) (Table 2). The combination of ESBL-encoding genes (78.1%; n = 25) was more frequent than the presence of one gene encoding for an enzyme alone (21.9%; n = 7). Among the CTX-M-producing *K. pneumoniae* isolates, all were positive for CTX-M group 1 (Table 2).

No significant association was found between the MAR index and the presence of *bla*_CTX-M_ (*p* = 0.54) and *bla*_TEM_ (*p* = 0.90). A tendency for a positive correlation was observed between the MAR index and the presence of *bla*_SHV_ (*p* = 0.08).

### 2.4. DNA Fingerprinting

**Figure 3:**
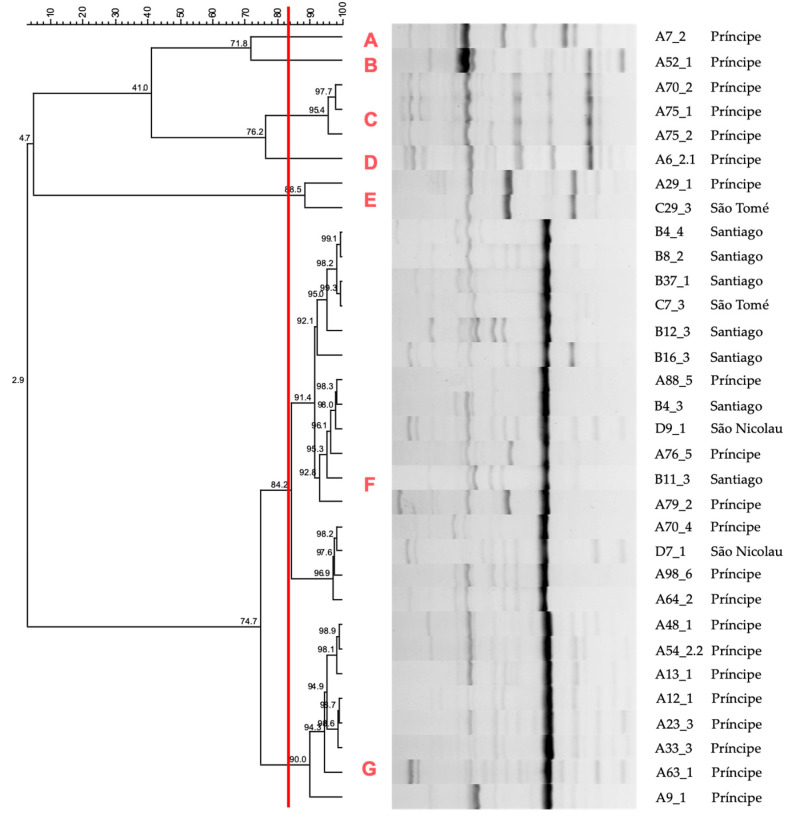
Dendrogram illustrating genomic variability by the ERIC-PCR typing of the 32 ESBL-producing *K. pneumoniae* isolates under study, displaying seven clusters (A–G). The cut-off line at 83.6% (determined reproducibility level) is shown in red.

The size of the bands obtained by PCR fingerprinting ranged from ≈120 bp to ≈1.6 kb. The relationships between the strains were based on fingerprinting profiles (Figure 3). In a global analysis, based on the cut-off defined by the reproducibility level (83.6%), seven clusters were identified, three of which were single-member clusters (A, B, and D), while the others consisted of two to sixteen isolates. The three isolates in cluster C, obtained from Príncipe, represent a single strain. The two isolates in cluster E correspond to a single strain, despite being obtained from animals from both Príncipe and São Tomé islands. Cluster F consists of 16 isolates from four different regions, but due to the similarity between them being greater than 83.6%, they represent a single strain. Cluster G, formed by eight isolates from animals in Príncipe, represents a single strain (the minimum similarity level between the isolates is 90%).

## 3. Discussion

This study reports that dogs from Cape Verde (Santiago and São Nicolau) and São Tomé and Príncipe can carry MDR ESBL-producing *K. pneumoniae* in their intestinal microbiota, posing a public health concern. *K. pneumoniae* is a major reservoir and vector for the transmission of significant AMR genes [29]. In Africa, it is the bacterial pathogen most frequently responsible for deaths attributable to AMR [30].

Given the public health implications of MDR ESBL-producing *K. pneumoniae*, it is necessary to understand its resistance mechanisms. Since the isolates under study were confirmed ESBL producers, broad resistance to cephalosporins was expected, as ESBL production is characterized by increased hydrolysis of oxyimino-β-lactams and inhibition by β-lactamase inhibitors. Aztreonam resistance was also expected, given that ESBL enzymes can inactivate this antimicrobial [4]. The reduction in susceptibility observed against combinations of β-lactams/β-lactamase inhibitors can be explained by the co-production of different β-lactamases, such as TEM and SHV variants. These have been previously reported to confer resistance to clavulanic acid and tazobactam, particularly variants such as TEM-30, TEM-31, TEM-50, TEM-121, and SHV-10 [4]. Notably, the isolates under study displaying intermediate susceptibility or non-susceptibility to β-lactam/β-lactamase inhibitor combinations (43,8%; n = 14) demonstrated a high rate of co-occurrence of *bla*_CTX-M_, *bla*_TEM_, and *bla*_SHV_ (92.9%; n = 13) or *bla*_CTX-M_ and *bla*_TEM_ (7.1%; n = 1), supporting the hypothesis that this co-production could be responsible for the observed pattern.

Beyond β-lactam resistance, ESBL-producing bacteria frequently express resistance to other antibiotic classes. The co-occurrence of resistance to non-β-lactam antibiotics among ESBL producers has already been reported. Studies indicate that ESBL-producing bacteria frequently exhibit a MDR profile [17] and a higher prevalence of resistance to tetracycline, fluoroquinolones, aminoglycosides, and trimethoprim/sulfamethoxazole when compared to non-ESBL producers [1,31]. A direct comparison between ESBL-producing and non-ESBL-producing isolates was not performed in our study, but the ESBL producers analyzed also showed high levels of resistance to tetracyclines (62.5%), ciprofloxacin (93.8%), gentamicin (50%), and trimethoprim/sulfamethoxazole (90.6%).

The MDR nature of ESBL-producing *K. pneumoniae* can be associated with the fact that ESBLs are plasmid-mediated enzymes which often carry multiple resistance genes via transposons or integrons, facilitating the occurrence of horizontal transfer to other bacteria, even across different species. Additionally, bacteria harboring multiple antibiotic resistance genes have been described to be widely disseminated in the community, further limiting therapeutic options in case of disease [1]. This demonstrates the role of mobile genetic elements in the persistence of MDR bacteria in the community, in the environment, and in healthcare sectors, making AMR control even more challenging. In this context, the need for last-resort antimicrobial compounds is urgent to effectively treat infections caused by these increasingly prevalent MDR isolates [32].

Consistent with another report on ESBL-producing bacteria [33], the isolates under study exhibited a low resistance rate to chloramphenicol (9.4%), suggesting that this antibiotic could serve as a reserve agent in these geographical locations for critical patients with ESBL infections. Regarding resistance against nitrofurantoin, it is reported that the prevalence of resistance against this antimicrobial compound does not significantly differ between ESBL and non-ESBL isolates [2] and that nitrofurantoin maintains good efficacy against ESBL-producing bacteria [34]. However, in our study, a notably high resistance rate to this antimicrobial was observed (53.1%), which raises concerns about its reliability as a treatment option. Nitrofurantoin resistance could be an indicator of the XDR phenotype among Enterobacteriaceae, harboring multiple AMR and efflux pump genes [35]. The increasing use of nitrofurantoin in combination therapy to treat infections caused by MDR bacteria with resistance to last-resort antibiotics, like carbapenems [36], makes this finding particularly concerning. Carbapenems have become the standard treatment for ESBL-producing Enterobacteriaceae [37], and fortunately, no resistance was detected against these antimicrobials in the isolates under study, contrarily to other reports on human isolates from Santiago [38] and São Tomé [39], suggesting that these strains do not circulate in animals as much as in humans.

Screening for three β-lactamase-encoding genes—*bla*_CTX-M_, *bla*_TEM_, and *bla*_SHV_—revealed their presence in 96.9%, 56.3%, and 65.6% of the isolates, respectively. The three genes were found in isolates from the four locations. These findings indicate that CTX-M enzymes are widely prevalent, while TEM and SHV occur at lower frequencies. Historically, TEM and SHV enzymes were predominant among β-lactamase-producing Enterobacteriaceae, but the extensive worldwide dissemination of CTX-M enzymes has led to a “CTX-M pandemic”, in which CTX-M has largely replaced other ESBL types among Enterobacteriaceae [40]. Additionally, since only one pair of primers was used for the detection of each gene, the protocols applied may fail to detect variants with sequence variations. Nevertheless, our findings demonstrate that TEM and SHV remain highly prevalent, often co-occurring with CTX-M in the same isolates. This persistence and co-presence are particularly concerning, making most β-lactam antibiotics, except for carbapenems, ineffective as treatment options for severe infections caused by ESBL-producing bacteria [41]. In fact, it would be relevant to further characterize the subtypes of SHV and TEM present in the isolates under study, as there is a significant difference in resistance patterns within these variants. These patterns range from a similar hydrolysis of benzylpenicillin and early cephalosporins, such as for TEM-1, TEM-2, and SHV-1 [4], to an ESBL pattern, such as for TEM-50, TEM-121, SHV-12, and SHV-18 [4,13]. Given that 21.9% of the isolates were positive for the presence of one β-lactamase-encoding gene, 37.5% for the presence of two genes, and 40.6% for the presence of three genes, it can be observed that the presence of more than one gene was more frequent than the presence of only a single gene. The co-existence of multiple β-lactamase genes can potentially enhance resistance levels, complicating treatment strategies. The observation of a trend toward a positive association between the presence of *bla_SHV_* and the MAR index (*p* = 0.08) suggests that isolates carrying this gene tend to have a higher MAR index. However, a study with a larger number of isolates and the sequencing of the SHV-encoding gene is needed to clarify this relationship.

Although this study focused on analyzing strains exhibiting a resistance pattern consistent with the presence of ESBL enzymes, the three isolates obtained during ESBL screening that tested negative in the phenotypic ESBL test were also subjected to AST and PCR for *bla*_TEM_, *bla*_SHV_, and *bla*_CTX-M_. The presence of *bla*_TEM_ was detected in two of these isolates, and AST revealed susceptibility to all tested antimicrobials. TEM enzymes typically lead to the hydrolysis of cephalosporins [4], which was not observed in these cases. The presence of this gene in the two isolates phenotypically negative for ESBL production must be interpreted cautiously. As previously mentioned, not all TEM enzymes exhibit ESBL activity. Since the TEM gene present in these isolates was not sequenced, it is not possible to conclude that it is responsible for resistance to the antibiotics present in the culture medium (third-generation cephalosporins). However, this resistance could also be explained by the existence of silent genes that were not expressed during ESBL phenotype confirmation and AST, a phenomenon already reported by other authors [42]. This raises concerns about the phenotypic detection of ESBL in Enterobacteriaceae, as the silencing of these genes can potentially be reversed [43] and, in such cases, may compromise treatment efficacy [44], highlighting the need for genotypic surveillance.

Given the high prevalence of MDR *K. pneumoniae*, it is crucial to investigate their clonal relatedness to better understand dissemination dynamics. To explore the potential dissemination of MDR *K. pneumoniae* across the studied locations, ERIC-PCR typing was performed, allowing the mapping of isolates according to their origin. A clustering pattern was observed, making it possible to identify seven clusters. All isolates from Santiago clustered within cluster F, though this cluster also contained isolates from all other locations. The two isolates from São Nicolau, Cape Verde, were exclusively associated with cluster F, suggesting a common origin between the isolates from the Cape Verde islands. Among the isolates from São Tomé, one was assigned to cluster F, while the other belonged to cluster E, indicating some genomic variability. The isolates from Príncipe exhibited the greatest genetic diversity, being distributed across all identified clusters. This may be related to the fact that the bacterial collection under study includes a higher number of isolates from animals from this location. Additionally, some clusters were unique to Príncipe, including single-member clusters A, B, and D, as well as cluster G. These findings confirm that the MDR *K. pneumoniae* isolates under study are genetically diverse.

There was no direct correlation between ERIC profiles and antimicrobial susceptibility patterns. Isolates with identical antibiotypes often had different ERIC profiles, while many isolates with distinct susceptibility patterns shared identical ERIC patterns, consistent with findings from other studies [45]. The data further suggest both the local expansion of specific clones and the interregional spread of identical strains, underscoring the dissemination potential of these resistant bacteria. This highlights the urgent need for continuous surveillance and molecular epidemiological studies to monitor the spread of MDR *K. pneumoniae*.

One of the most important questions raised by these findings is of the origin of the resistant bacteria present in the dogs’ microbiota. It is well established that antimicrobial use is a major driver of AMR development, as it creates selective pressure that promotes the survival of resistant strains [46]. Unfortunately, information on previous antimicrobial treatments in these animals was not available, which represents a limitation of this study. However, to the best of the authors’ knowledge, there are no data on the use of cephalosporins or other relevant antibiotics to treat infections in humans or dogs in these locations. However, given that some of the sampled animals were stray dogs, it is unlikely that they had prior exposure to antimicrobial therapy. In fact, no association was observed between the MAR index of the isolates and ownership status. These dogs are likely acquiring resistant bacteria from external sources, such as food, water, other animals, humans, or even the environment. In fact, in resource-limited settings with inadequate sanitation, the disposal of human and animal waste facilitates the dissemination of resistant bacteria into surface water [47] and the environment, which may reach both human and animal populations. Consequently, AMR often reflects anthropogenic activities, and the close interaction between the three One Health sectors leads to multiple routes of AMR dissemination, potentially contributing to the emergence of AMR hotspots [48]. However, the scarcity of data on the epidemiology of the dog population examined hinders the understanding of the origin and spread of AMR within the environment of the companion animals. Regardless of the exact origin of AMR, these findings raise public health concerns, given the potential for dog-to-human transmission and the contamination of the surrounding environment with resistant *K. pneumoniae*, contributing to the persistence and spread of MDR ESBL-producing bacteria in the studied areas. A larger-scale study, incorporating human and environmental sampling, would be essential to fully understand the dissemination of such bacteria in these communities [49]. A study by Tängdén et al. [50] further reinforces these concerns, demonstrating that traveling to areas with a high prevalence of ESBL-producing strains represents a high-risk factor for colonization, suggesting that local hotspots of AMR may not only impact the resident population but also pose a health risk to travelers, accelerating the widespread dissemination of these resistance genes.

There is a high scarcity of data on AMR in Cape Verde, with only two studies reporting ESBL-producing Enterobacteriaceae in the human clinical setting [38] and in companion and stray dogs [51]. Regarding the human healthcare setting, a study conducted in a hospital on Santiago Island screened inpatients for rectal colonization by ESBL-producing bacteria [38]. The study reported a high frequency (56%, n = 55) of ESBL-producing Enterobacteriaceae, mainly *K. pneumoniae* (58%) and *E. coli* (37%), and detected five different ESBLs, with CTX-M-15 being highly predominant (92%), and SHV-2 (2%) being detected only once in a *K. pneumoniae* isolate. Our study also detected a high frequency of CTX-M (96.9%; n = 31), with all members belonging to CTX-M group 1, which includes CTX-M-15. Also, the detection of genes encoding for TEM (71.4%; n = 5) and SHV (100.0%; n = 7) in isolates from Santiago reveals that these genes are still circulating in dogs from this island. In the same location, a study by Monteiro et al. [52] reported a high frequency of cephalosporin resistance in humans. However, ESBL presence was not investigated. Regarding ESBL detection in animal isolates, the available information is also very limited. The only study available is by Matos et al. [51], reporting the high-rate detection (29.0%; n = 29) of ESBL-producing Enterobacteriaceae in dogs, with 13.6% being *K. pneumoniae*, which is in accordance with our results. However, this study—as well as any other available data—does not describe β-lactamase-encoding genes circulating in animals from this area. Therefore, this study presents for the first time the presence of these genes in dogs from Santiago.

In São Tomé and Príncipe, data on AMR are even more limited. In a study from 2018 [39], 50 patients were screened for MDR Enterobacteriaceae, in which only CTX-M-15 (CTX-M group 1)- and TEM-1-producing *K. pneumoniae* strains were detected, in accordance with our results. Even though only two of the isolates from our study were from this location, SHV was detected once, revealing that SHV enzymes are still circulating in São Tomé. As far as the authors are aware, there are no studies available on AMR on Príncipe Island in humans, animals, or the environment.

Overall, the antimicrobial susceptibility profiles of the isolates under study raise concerns due to their high resistance rate against antimicrobials frequently used in both human and veterinary medicine. This highlights the urgent need for continuous surveillance and the development of alternative strategies to combat the rising threat of multidrug-resistant ESBL-producing *K. pneumoniae*.

To the best of the authors’ knowledge, this is the first study on the detection and identification of ESBL-encoding genes present in isolates from animals from Cape Verde and São Tomé and Príncipe. The relevance and role of AMR dissemination transcend the human context and are closely related to the human–animal–environment interface, with dogs acting as a suitable bridge for the interspecies transmission of antimicrobial-resistant bacteria due to their direct and indirect contact with humans [53]. This highlights the need for a One Health approach and provides valuable data on AMR trends, with the aim of mitigating the spread of these resistant strains.

## 4. Materials and Methods

### 4.1. Sample Collection

**Figure 4:**
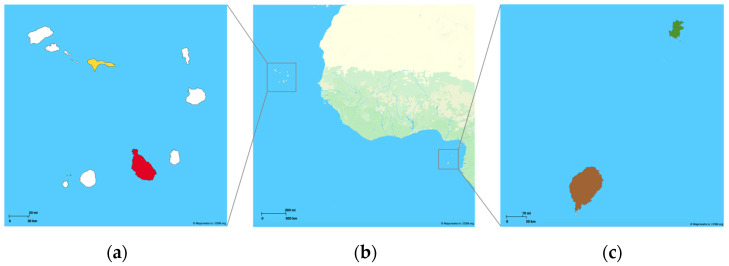
Map of the study area and location of the sampling sites. The image on the left (**a**) represents the Cape Verde archipelago, highlighting Santiago (red) and São Nicolau (yellow); image (**b**) shows the West African coast where the study sites are located; the image on the right (**c**) represents the São Tomé (brown) and Príncipe (green) islands. Mapcreator.io. (https://mapcreator.io, accessed on 14 March 2025).

Between December 2022 and March 2023, 195 rectal swabs were collected from dogs across four islands of two different African countries along the western coast, including São Tomé (35 swabs, 18.0% of the total samples), Príncipe (96 swabs, 49.2%), Santiago (45 swabs, 23.1%), and São Nicolau (19 swabs, 9.7%) (Figure 4). The samples were collected as part of a campaign led by Veterinary Without Borders Portugal, which focused on the neutering and deworming of dogs in these African countries. Amies swabs (VWR^TM^, Leuven, Belgium) were used for sample collection, being stored refrigerated until further processing in the Microbiology Laboratory of the Faculty of Veterinary Medicine, University of Lisbon, Portugal.

A randomly selected subset of dogs participating in this campaign was included in the study, regardless of whether they were companion animals or stray dogs. There were no restrictions based on gender, age, or health status. Detailed information on the dogs’ location, age, gender, and ownership status can be found in the Supplementary Files (Table S1).

All animals were cared for according to the rules set by the current EU (Directive 2010/63/EC) and Portuguese legislation (DL 113/2013), by the competent authority in Portugal (https://www.dgav.pt/animais, accessed on 22 January 2025), and by Cape Verde (Penal Code Regulatory Decree No. 12/2020) and São Tomé and Príncipe (Law No. 13/2005) legislation. Only noninvasive samples were collected during routine procedures, and no ethics approval was needed. Trained veterinarians obtained all samples, following standard routine procedures. No animal experiments were performed. In the case of companion animals, verbal informed consent was obtained from all owners, with all necessary information about the study being provided before obtaining participants’ consent.

### 4.2. Bacterial Isolation

Swabs were inoculated into 5 mL of Brain Heart Infusion (BHI) enrichment broth (VWR^TM^, Lisbon, Portugal) at 37 °C for 24 h under aerobic conditions. A 10 µL loopful of the broth culture was then streaked onto chromID^®^ ESBL agar plates (bioMérieux, Marcy-l’Étoile, France), a selective chromogenic medium designed for the isolation and detection of ESBL, based on a rich nutrient agar supplemented with a mixture of antibiotics, including cefpodoxime [54]. Plates were incubated at 37 °C for 18–24 h under aerobic conditions. Greenish blue colonies were further isolated onto MacConkey agar (Oxoid, Hampshire, UK) plates to assess lactose fermentation, and further isolated onto BHI agar, prepared by supplementing BHI broth with 1.4% bacteriological agar (VWR^TM^, Leuven, Belgium), applying the same incubation conditions, prior to their characterization through Gram staining, microscopic morphology observation, and oxidase reaction. In summary, isolates that produced greenish blue colonies on chromID^®^ ESBL and lactose-fermenting colonies on MacConkey agar, and were Gram-negative and oxidase-negative were presumptively identified as *K. pneumoniae*. The isolates were stored in buffered peptone water (VWR^TM^, Leuven, Belgium) supplemented with 20% glycerol (VWR^TM^, Lisbon, Portugal) at −20 °C until further use. *K. pneumoniae* CECT 7787 and *E. coli* ATCC 25922 strains were used as positive and negative controls of growth on chromID^®^ ESBL agar, respectively.

### 4.3. Bacterial Identification

All isolates were identified at the species level using matrix-assisted laser desorption/ionization time-of-flight mass spectrometry (MALDI-TOF MS) analysis (Bruker Daltonics GmbH, Bremen, Germany). Briefly, bacterial biomass obtained from colonies grown overnight was applied in a steel target plate (Bruker Daltonics GmbH, Bremen, Germany) and overlaid with 1 μL of α-cyano-4-hydroxycynnamic acid–matrix solution (HCCA, Bruker Daltonics GmbH, Bremen, Germany). The acquisition and analysis of the results were carried out by a Bruker Daltonics Autoflex Speed spectrometer (Bruker Daltonik GmbH, Bremen, Germany). For quality control, the instrument was calibrated using BTS (Bruker Daltonik GmbH, Bremen, Germany). The mass spectra were generated by MBT Compass Explorer software v4.1 (Bruker Daltonik GmbH, Bremen, Germany), which compares each sample mass spectrum to the reference mass spectra in the available databases. Standard interpretative criteria were applied, and scores of ≥2.0 were accepted for species assignment.

### 4.4. Confirmation of the ESBL Phenotype

The ESBL phenotype of *K. pneumoniae* isolates was assessed following Clinical and Laboratory Standards Institute (CLSI) standards [55]. Briefly, approximately one colony of each isolate was suspended in a tube containing 3 mL of 0.85% NaCl, to a final turbidity of 0.5 McFarland. Immediately after, each inoculum was spread on Mueller Hinton agar (Oxoid, Hampshire, UK) using a sterile swab. Disks (Oxoid, Hampshire, UK) of ceftazidime (30 µg), cefotaxime (30 µg), ceftazidime–clavulanate (30/10 µg), and cefotaxime–clavulanate (30/10 µg) were placed on the agar surface. Disks of ceftazidime–clavulanate and cefotaxime–clavulanate were prepared according to CLSI standards [55] and used immediately. Plates were incubated at 37 °C for 16–18 h. After, a strain was classified as an ESBL-producing strain when a ≥5 mm increase was observed in the inhibition zone diameter for the antimicrobial agent tested in combination with clavulanate vs. the zone diameter of the agent when tested alone. *K. pneumoniae* CECT 7787 and *E. coli* ATCC 25922 were used as positive and negative controls, respectively. A 10% replica was performed.

### 4.5. Antimicrobial Susceptibility Testing

All confirmed ESBL-producing *K. pneumoniae* isolates were subjected to antimicrobial susceptibility profiling toward 24 antimicrobials using the Kirby–Bauer disk diffusion method [56]. Briefly, a bacterial suspension was performed as described in the previous section, and immediately after, each inoculum was spread on the surface of Mueller Hinton agar plates using a sterile swab. Antibiotic disks (Oxoid, Hampshire, UK) were placed on the agar surface, and plates were incubated at 37 °C for 16–18 h. The antimicrobials selected for testing are representative of important classes of antimicrobial drugs used in both veterinary and human medicine, and included the following: cephalosporins (cefpodoxime 10 μg, cefotaxime 30 μg, ceftazidime 30 μg, cefepime 30 μg, and ceftaroline 30 μg); cephamycins (cefoxitin 30 μg); monobactams (aztreonam 30 μg); β-lactam combination agents (amoxicillin/clavulanate 20/10 μg and piperacillin/tazobactam 100/10 μg); carbapenems (meropenem 10 μg and imipenem 10 μg); aminoglycosides (gentamicin 10 μg), tetracyclines (tetracycline 30 μg and doxycycline 30 μg); fluoroquinolones (ciprofloxacin 5 μg); folate pathway antagonists (trimethoprim/sulfamethoxazole 1.25/23.75 μg); nitrofurans (nitrofurantoin 30 μg); and phenicols (chloramphenicol 30 μg). A 10% replica was performed. After incubation, the inhibition zones were measured, and isolates were categorized as susceptible (S), intermediate (I), or resistant (R) according to CLSI standards using human clinical breakpoints, as this study is within the One Health context and includes antimicrobials used only in humans [55]. *E. coli* ATCC 25922 and *P. aeruginosa* ATCC 27853 were used to validate the results. Expert rules were not used for the interpretation of antimicrobial susceptibility testing because they were not applicable. Isolates were classified as multidrug-resistant (MDR) when they presented resistance to ≥1 agent in ≥3 antimicrobial classes [28]. The multiple antibiotic resistance (MAR) index was also calculated for each isolate, being calculated as the ratio of the number of antibiotics an isolate was resistant to, compared to the total number of antibiotics tested, according to Singh et al. [57].

### 4.6. DNA Extraction

The total DNA of each isolate was extracted using the boiling method [58]. Briefly, a loopful of growth colonies was resuspended in 100 μL of 1× Tris–EDTA (TE) buffer solution (VWR, Philadelphia, PA, USA) supplemented with 0.1% Tween 20 (Fisher Bioreagents, Pittsburgh, MA, USA), incubated at 100 °C for 10 min, and further centrifuged at 18,800× *g* for another 10 min. The supernatant was collected, and DNA concentration and purity were assessed using a NanoDrop™ Spectrophotometer (ThermoFisher Scientific, Waltham, MA, USA). Subsequently, DNA suspensions were diluted in PCR-grade water (VWR, Philadelphia, PA, USA) to obtain a standardized concentration of 50 ng/μL and kept at −20 °C until further use.

### 4.7. Identification of β-Lactamase Genes by Multiplex PCR

Isolates were screened for the presence of three β-lactamase genes, *bla*_SHV_, *bla*_TEM_, and *bla*_CTX-M_, using a multiplex PCR adapted from Monstein et al. [59]. PCR was carried out in a reaction volume of 20 µL, which included the primers represented in Table 3 (STABVIDA, Caparica, Portugal), each at a final concentration of 0.4 μM, 10 µL of Supreme NZYTaq II 2× Green Master Mix (NZYtech, Lisbon, Portugal), 100 ng of DNA template, and 3.2 µL of PCR-grade water. The thermocycler (VWR, Radnor, PA, USA) conditions applied consisted of an initial denaturation at 95 °C for 5 min, followed by 30 cycles of denaturation at 94 °C for 30 s, annealing at 60 °C for 30 s, and extension at 72 °C for 2 min, with a final extension at 72 °C for 10 min.

The strains in which the presence of *bla*_CTX-M_ was detected were further analyzed by PCR using primers specific for the amplification of *bla*_CTX-M_ groups 1, 2, 8, 9, and 25, adapted from Woodford [60]. PCR was carried out in a reaction volume of 20 µL, which included the primers represented in Table 3 (STABVIDA, Caparica, Portugal), at a final concentration of 0.327 μM, 0.25 µL of PCRBIO HS Taq DNA Polymerase (PCR Biosystems, London, UK), 4 µL of 5× PCRBIO reaction buffer (PCR Biosystems, London, UK), 100 ng of DNA template, and 6.7 µL of PCR-grade water. The thermocycler (VWR, Radnor, PA, USA) conditions used consisted of an initial denaturation at 94 °C for 5 min, followed by 30 cycles of denaturation at 94 °C for 25 s, annealing at 52 °C for 40 s, and extension at 72 °C for 50 s, ending with a final extension step at 72 °C for 6 min. A 10% replica was performed.

All PCR products were revealed through electrophoresis, using a 1.5% agarose (NZYtech, Lisbon, Portugal) gel prepared with 1× Tris–Borate–EDTA (TBE) buffer solution and stained with 0.005% of Green Safe (NZYtech, Lisbon, Portugal). Ladder VI (50–1500 bp) (NZYtech, Lisbon, Portugal) was used as a molecular weight marker, and electrophoresis was run at 70 V for 1 h 30 min. The bands were visualized under ultraviolet radiation using the Bio-Rad ChemiDoc XRS imaging system (Bio-Rad Laboratories, Hercules, CA, USA). As positive control strains, *Enterobacter hormaechei* subsp. *hoffmannii* CCUG 58962, producing CTX-M group 1 and TEM, and *K. pneumoniae* CECT 7787, producing SHV, were used. Two negative controls were used for both reactions: *E. coli* ATCC 25922, which does not produce ESBL, and PCR-grade water.

**Table 3:** Primer sequences (5′–3′) used for the determination of the presence of *bla*_CTX-M_, *bla*_TEM_, and *bla*_SHV_, as well as *bla*_CTX-M_ groups 1, 2, 8, 9, and 25, and expected amplicon sizes.

Target		Primer Sequence (5′–3′)	Amplicon Size (bp)
*bla*_SHV_	Forward	ATG CGT TAT ATT CGC CTG TG	747
	Reverse	TGC TTT GTT ATT CGG GCC AA	
*bla*_TEM_	Forward	TCG CCG CAT ACA CTA TTC TCA GAA TGA	445
	Reverse	ACG CTC ACC GGC TCC AGA TTT AT	
*bla*_CTX-M_ ^1^	Forward	ATG TGC AGY ACC AGT AAR GTK ATG GC	593
	Reverse	TGG GTR AAR TAR GTS ACC AGA AYC AGC GG	
*bla*_CTX-M1_	Forward	AAA AAT CAC TGC GCC AGT TC	415
	Reverse	AGC TTA TTC ATC GCC ACG TT	
*bla*_CTX-M2_	Forward	CGA CGC TAC CCC TGC TAT T	522
	Reverse	CCA GCG TCA GAT TTT TCA GG	
*bla*_CTX-M8_	Forward	TCG CGT TAA GCG GAT GAT GC	666
	Reverse	AAC CCA CGA TGT GGG TAG C	
*bla*_CTX-M9_	Forward	CAA AGA GAG TGC AAC GGA TG	205
	Reverse	ATT GGA AAG CGT TCA TCA CC	
*bla*_CTX-M25_	Forward	GCA CGA TGA CAT TCG GG	327
	Reverse	AAC CCA CGA TGT GGG TAG C	

^1^ This reaction uses degenerate primers, where certain positions can vary to allow the amplification of different variants of the target sequence, presenting Y, R, and K degenerate nucleotides. In a degenerate primer, Y corresponds to a pyrimidine (C or T), R corresponds to a purine (A or G), and K corresponds to G or T, according to the International Union of Pure and Applied Chemistry (IUPAC) nucleotide code [61].

### 4.8. DNA Fingerprinting

The DNA fingerprinting of isolates was assessed through enterobacterial repetitive intergenic consensus (ERIC) PCR, adapted from Cabral et al. [6], using an ERIC2 primer (5′ AAG TAA GTG ACT GGG GTG AGC G 3′). PCR was carried out in a reaction volume of 25 µL, which included 0.25 µL of PCRBIO VeriFi^TM^ Polymerase (PCR Biosystems, London, UK), 5 µL of 5× PCRBIO VeriFi^TM^ Buffer (PCR Biosystems, London, UK), 0.4 μM of primer, 100 ng of genomic DNA, and 16.75 µL of PCR-grade water. The amplification protocol included an initial denaturation cycle at 95 °C for 3 min, followed by 40 cycles of denaturation at 92 °C for 1 min, annealing at 36 °C for 1 min, extension at 72 °C for 1 min, and a final step at 72 °C for 16 min. PCR products were loaded on 1.5% agarose gel with 0.005% Green Safe at a constant voltage of 70 V for 2 h 30 min in 1× TBE buffer. Ladder VII (100–3000 bp) (NZYtech, Lisbon, Portugal) was used as a molecular weight marker. The gels were visualized as described in the previous section. The profiles were compared using BioNumerics^®^ 6.6 (Applied Maths, Kortrijk, Belgium) with a hierarchical numerical process based on the Pearson correlation coefficient (without optimization) and the unweighted pair group method with arithmetic mean (UPGMA) for agglomerative clustering. A 10% replica was performed, and the reproducibility value was determined as the average value of four pairs of isolates.

### 4.9. Data Analysis

Microsoft Excel (Microsoft Corporation, Redmond, WA, USA) was used to determine the frequencies, and further data analysis was performed using SAS software version 9.4 (SAS Institute Inc., Cary, NC, USA). Given the sample size and the nature of the MAR index response variable, the Mann–Whitney U test (PROC NPAR1WAY) was used to test the association between MAR index with ownership status of the sampled dogs and the presence of *bla*_CTX-M_, *bla*_TEM_, and *bla*_SHV_. Differences were considered significant when *p* ≤ 0.05, whereas a tendency was defined as 0.05 < *p* ≤ 0.10.

## 5. Conclusions

The results from our study provide insights into the high prevalence of *K. pneumoniae* resistance to third-generation cephalosporins, as well as to other important antimicrobial groups, such as fluoroquinolones and tetracyclines, in dogs from two islands of Cape Verde (Santiago and São Nicolau) and São Tomé and Príncipe. The results also revealed the prevalence of three important *bla* genes, encoding SHV, TEM, and CTX-M, in the ESBL-producing *K. pneumoniae* isolates under study. The results also suggest that there is a possible dissemination of these strains between the archipelagos of Cape Verde and São Tomé and Príncipe, possibly caused by tourism or migratory movements. However, fingerprinting through ERIC did not allow a definitive conclusion about the genetic relatedness between these isolates, which could be obtained through MLST or PFGE analysis. Further studies are required to determine if these isolates also circulate in humans and in the environment. This study suggests a need to strengthen AMR surveillance and to strengthen laboratory capacity for antimicrobial susceptibility testing in these locations.

## Figures and Tables

**Table 1:** Resistance profile of the *K. pneumoniae* isolates under study against all tested antimicrobials, displayed in percentages (n = 32). G—generation; S—susceptible; I—intermediate; R—resistant.

Antimicrobial Class	Antimicrobial	S (%)	I (%)	R (%)
3rd-G cephalosporins	Ceftazidime	0.0	15.6	84.4
	Cefotaxime	0.0	0.0	100.0
	Cefpodoxime	0.0	0.0	100.0
4th-G cephalosporins	Cefepime	0.0	6.3	93.8
5th-G cephalosporins	Ceftaroline	0.0	0.0	100.0
Cephamycins	Cefoxitin	100.0	0.0	0.0
Monobactams	Aztreonam	0.0	0.0	100.0
β-Lactam combination agents	Amoxicillin/clavulanate	53.6	31.3	12.5
	Piperacillin/tazobactam	90.6	0.0	9.4
Carbapenems	Imipenem	100.0	0.0	0.0
	Meropenem	100.0	0.0	0.0
Aminoglycosides	Gentamicin	15.6	34.4	50.0
Tetracyclines	Tetracycline	37.5	0.0	62.5
	Doxycycline	37.5	0.0	62.5
Fluoroquinolones	Ciprofloxacin	6.3	0.0	93.8
Folate pathway antagonists	Trimethoprim/sulfamethoxazole	9.4	0.0	90.6
Phenicols	Chloramphenicol	84.4	6.3	9.4
Nitrofurans	Nitrofurantoin	21.9	25.0	53.1

**Table 2:** Frequency of β-lactamase-encoding genes and β-lactamase distribution patterns in the ESBL-producing *K. pneumoniae* isolates under study (n = 32).

	Enzyme(s)	Frequency (%)
β-lactamase presence	CTX-M	96.9
	TEM	56.3
	SHV	65.6
CTX-M groups	CTX-M1	96.9
	CTX-M2	0.0
	CTX-M8	0.0
	CTX-M9	0.0
	CTX-M25	0.0
β-lactamase distribution patterns	CTX-M	18.8
	TEM	0.0
	SHV	3.1
	CTX-M + TEM	15.6
	CTX-M + SHV	21.9
	TEM + SHV	0.0
	CTX-M + TEM + SHV	40.6

## Data Availability

The data presented in this study are available in the article and Supplementary Materials. More details can be provided upon reasonable request to the correspondence contacts.

## Supplementary Materials

The following supporting information can be downloaded at https://www.mdpi.com/article/10.3390/antibiotics14040408/s1, Table S1: Information on the location, gender, age, and ownership status of the dog samples from which the *Klebsiella pneumoniae* isolates under study were obtained.
