# Supplementary material for: Extended-Spectrum β-Lactamase-Producing Klebsiella pneumoniae in Dogs from Cape Verde and São Tomé and Príncipe: Implications for Public Health
Source: Antibiotics (Basel). 2025 Apr 16;14(4):408. doi: 10.3390/antibiotics14040408 (PMC12023937; doi:10.3390/antibiotics14040408)
Supplement: Supplementary file 1 [file antibiotics-14-00408-s001.zip › antibiotics-3578500-supplementary.pdf]

Table S1. Information on the location, gender, age, and ownership status of the dog samples from which the *Klebsiella pneumoniae* isolates under study were obtained.

Location	Sample	Isolate	Gender	Age	Ownership status
Príncipe	A6	A6_2.1	Male	<5 years	Pet
	A7	A7_2	Male	<5 years	Pet
	A9	A9_1	Female	>5 years	Stray
	A12	A12_1	Female	Indetermined	Stray
	A13	A13_1	Female	<6 months	Pet
	A23	A23_3	Male	Indetermined	Stray
	A29	A29_1	Female	>5 years	Pet
	A33	A33_3	Male	>5 years	Pet
	A48	A48_1	Male	Indetermined	Indetermined
	A52	A52_1	Female	<6 months	Indetermined
	A54	A54_2.2	Female	>5 years	Indetermined
	A63	A63_1	Female	Indetermined	Stray
	A64	A64_2	Female	Indetermined	Pet
	A70	A70_2	Male	<5 years	Pet
		A70_4			
	A75	A75_1	Male	<5 years	Indetermined
		A75_2			
	A76	A76_5	Male	>5 years	Pet
	A79	A79_2	Male	Indetermined	Pet
	A88	A88_5	Female	<6 months	Pet
	A98	A98_6	Female	Indetermined	Stray
Santiago	B4	B4_3	Male	<5 years	Stray
		B4_4			
	B8	B8_2	Male	<6 months	Indetermined
	B11	B11_3	Female	<5 years	Indetermined
	B12	B12_3	Female	<5 years	Stray
	B16	B16_3	Female	<5 years	Stray
	B37	B37_1	Female	Indetermined	Stray
São Tomé	C7	C7_3	Female	Indetermined	Stray
	C29	C29_3	Female	Indetermined	Stray
São Nicolau	D7	D7_1	Male	>5 years	Indetermined
	D9	D9_1	Male	<5 years	Indetermined
